# Inflammatory cytokines induce MAdCAM-1 in murine hepatic endothelial cells and mediate alpha-4 beta-7 integrin dependent lymphocyte endothelial adhesion *In Vitro*

**DOI:** 10.1186/1472-6793-7-10

**Published:** 2007-09-14

**Authors:** Tomoaki Ando, Robert R Langley, Yuping Wang, Paul A Jordan, Alireza Minagar, J Steven Alexander, Merilyn H Jennings

**Affiliations:** 1 Nagoya City Medical University, 1-Kawasumi-Mizuho, Nagoya, 467-8601, Japan; 2 Department of Cancer Biology, The University of Texas M D Anderson Cancer Center, Houston, Texas 77030, USA; 3 Department of Obstetrics and Gynecology, Louisiana State University Health Sciences Center, Shreveport, LA 71130, USA; 4 Department of Gastroenterology, Louisiana State University Health Sciences Center, Shreveport, LA 71130, USA; 5 Department of Neurology, Louisiana State University Health Sciences Center, Shreveport, LA 71130, USA; 6 Department of Molecular and Cellular Physiology, Louisiana State University Health Sciences Center, Shreveport, LA 71130, USA

## Abstract

**Background:**

MAdCAM-1 plays a central role in T-lymphocyte homing to the gut, but its role in chronic liver inflammation remains unknown. Therefore, this study measured MAdCAM-1 expression, regulation, and function in cultured murine hepatic endothelial cells.

**Methods:**

Cultures of hepatic endothelial cells (HEC) were prepared from mice expressing a temperature-sensitive SV40 large T antigen (*H-2K*^*b*^-*tsA58*) under the control of an IFN-γ promoter. Time and dose dependent expression of MAdCAM-1 in response to TNF-α, IL-1β and IFN-γ was studied by immunoblotting. Lymphocyte adhesion was studied using α_4_β_7_integrin expressing lymphocytes (TK-1) +/- anti-MAdCAM-1 mAb.

**Results:**

TNF-α induced MAdCAM-1 dose-and time-dependently with maximum expression at 20 ng/ml and at 48 hours. IL-1β also induced MAdCAM-1 to a lesser extent compared to TNF-α; IFN-γ did not induce MAdCAM-1. TNF-α significantly increased lymphocyte-endothelial adhesion (*P *< 0.01), which was reversed by anti-MAdCAM-1 antibody. MAdCAM-1 expression was also reduced by N-acetylcysteine and by two NO donors (SperNO, DETANO) suggesting that hepatic endothelial MAdCAM-1 is oxidant and NO regulated.

**Conclusion:**

MAdCAM-1 is a major determinant of leukocyte recruitment in chronic inflammation and is expressed by HEC in response to IL-1β and TNF-α. This system may provide a useful model for studying inflammatory mechanisms in liver disease and help determine if controlled MAdCAM-1 expression might influence inflammation in liver disease.

## Background

Lymphocyte trafficking to secondary lymphatic organs including peripheral lymph nodes, Peyer's patches, bone marrow, spleen, and mesenteric lymph nodes, is largely governed by adhesion molecules on lymphocytes, called *homing receptors*, and their corresponding ligands on endothelial cells, called *vascular addressins *[1]. Binding through these molecules increases the frequency with which these cell arrest on cells expressing these cognate antigens. Most memory and effector lymphocytes also access and recirculate through extra-lymphoid immune effector sites. The liver, like the skin and gut, is a major site of antigen presentation with its own resident lymphocyte population. On the other hand, at sites of inflammation, lymphocyte homing depends on interactions between lymphocytes and *venular *endothelial cells. It is becoming increasingly apparent that inflammation is associated with enhanced expression of endothelial cell adhesion molecules (ECAMs) in the intestines of both experimental animals and humans [2,3]. Liver endothelial cells have also been shown to express intercellular adhesion molecule-1 (ICAM-1), intercellular adhesion molecule-2 (ICAM-2), and vascular cell adhesion molecule-1 (VCAM-1) [4,5]. Recent studies indicate that in hepatic ischemia reperfusion injury, blocking ICAM-1 reduced inflammation and liver injury, and ICAM-1 immunoblockade has been considered as a possible target for therapeutic intervention [6]. However, the precise mechanism of adhesion and transmigration of lymphocytes in the hepatic vasculature is still poorly understood. Recently it was reported that MAdCAM-1 was expressed in the liver portal region in autoimmune hepatitis, and MAdCAM-1 mediated adhesion might provide a basis for *hepatic *recruitment of mucosal lymphocytes, (at least in inflammatory bowel disease complicated by liver disease) [7].

Mucosal addressin cell adhesion molecule-1(MAdCAM-1) is a ~ 60 kDa endothelial cell adhesion molecule expressed on the surface of high endothelial venules in the gut and in Peyer's patches [8]. MAdCAM-1 is also expressed on endothelial cells within the mesenteric lymph nodes, the lamina propria of both the small and large intestine, in the mammary gland during lactation and on brain endothelial cells [9,10]. In addition to its normal role in lymphocyte trafficking to mucosal lymphoid tissue, MAdCAM-1 expression is also dramatically increased in chronic inflammatory and disease states (33), e.g., inflammatory bowel disease (IBD) [2], sclerosing cholangitis, cirrhosis [12], and diabetes [13,14], and may play important roles in these conditions. In IBD, especially Crohn's disease, MAdCAM-1 acts as the main ligand for α_4_β_7_-expressing lymphocytes, and recruits these lymphocytes into the intestine where they initiate and sustain chronic inflammation. Several animal models and human studies support an absolute requirement for both MAdCAM-1 and α_4_β_7 _in the production of immune models of colitis [15].

MAdCAM-1 is expressed at the surface of lymphoid and brain endothelial cells in response to several cytokines including TNF-α and IL-lβ. However, the signal transduction pathways involved in MAdCAM-1 induction in HEC have not been studied. Since MAdCAM-1 is induced by Th1 cytokines, like TNF-α and IL-lβ, it is likely that its induction would mechanistically resemble that described for other adhesion molecules (such as ICAM-1 and VCAM-1). These adhesion molecules are also induced by Th1 cytokines, and require activation of the NF-kB/PARP complex [16]. Activation of these transcription factors is also believed to require the production of intracellular oxidants (since synthesis of these adhesion molecules in response to cytokines is prevented by antioxidants like N-acetylcysteine). Physiologically, the expression of these adhesion molecules and forms of injury mediated by them also appears to be limited by the presence of NO produced by either constitutive or inducible forms of nitric oxide synthase (eNOS, iNOS) or by NO donors [17-19]. It has been suggested that NO might limit the transcription/translation of adhesion molecules either by scavenging signal oxidants (produced in response to cytokines) or by covalent modification of polypeptides in the signaling pathway, like the inhibitor of kappaB (IkB).

In the intact liver, oxidative stress appears to be part of the mechanism for ischemia and reperfusion (I/R) injury and is closely related to the generation of reactive oxygen species (ROS) [20] and possibly nitric oxide [21]. Sinusoidal endothelial cells appear to be a major target of liver I/R in vivo as well as hypoxia-reoxygenation injury in vitro [22,23]. The I/R injury to sinusoidal endothelium disrupts the microcirculation, reducing blood flow and enhancing further tissue necrosis. Neutrophils will then adhere to damaged endothelial cells, and their subsequent activation is likely to be an important source of ROS. Endogenous levels of reduced glutathione (GSH), critically important in providing protection against injury from ROS during I/R injury in the intact liver [24] as well as in the other tissue [25], are reduced following I/R. The role of the hepatic endothelial cell in the production of autocrine oxidative stress remains unclear.

In the present study, we used a hepatic endothelial cell line from transgenic mice whose tissues maintain a temperature-sensitive SV40 large T antigen (H-2K^b^-tsA58 mice) to assess the MAdCAM-1 expression of HEC directly. Our data show that hepatic microvessel endothelial MAdCAM-1 is induced by TNF-α and IL-1β, but not by IFN-γ Expression of MAdCAM-1 was dose-dependently increased by TNF-α. Further, we showed that the HEC bound TK-1 lymphocytes to a significantly higher extent after TNF-α stimulation and that this adhesion was inhibited by anti-MAdCAM-1 antibody. We also evaluated whether endogenous nitric oxide (from eNOS and iNOS), exogenous NO (from rapid or slow-releasing NO donors) or redox imbalance affects the expression of MAdCAM-1. Our data suggest that in this model, insufficient NO may be produced directly by HEC (from either iNOS or eNOS), to influence MAdCAM-1 expression. However, these cells can respond to NO from other sources (rapid and slow releasing NO donors), and exogenous NO potently decreases both MAdCAM-1 expression and lymphocyte endothelial adhesion.

## Methods

### Cells

Hepatic microvascular endothelial cells were prepared according to Langley et al. [26]. Briefly, the cells were isolated from livers of mice, homozygous for a temperature-sensitive SV40 large T antigen (ImmortoMice; CBA/ca X C57Bl/10 hybrid; Charles River Laboratories). The culture medium was DMEM including 10% fetal bovine serum, 2-mM L-glutamine, nonessential amino acids, a vitamin solution and 1% antibiotic/anti-mycotic (Life Technologies, Inc., Rockville, MD). The culture flasks were coated by 2% gelatin. After the cultures reached confluence, the cells were maintained in the same medium. We cultured HEC under permissive temperatures (33°C) and transferred them to non-permissive temperatures (37°C) to inactivate SV40 large T antigen 24 h before cytokine stimulations.

### Western analysis of cell lysates

Protein samples (75 μg each) were separated on 7.5% SDS-PAGE and transferred to nitrocellulose membranes. Membranes were incubated with 1° anti-mouse MAdCAM-1 monoclonal antibody (mAb; 10 μg/ml MECA-367; Pharmingen, San Diego, CA). Goat anti-rat horseradish peroxidase-conjugated 2° antibody (Sigma) was added at a 1:2,000 dilution. Last, membranes were developed by enhanced chemiluminescence (Amersham, La Jolla, CA). MAdCAM staining density was measured by scanning the 58-to 60-kDa band and densitometry using Image Pro Plus (Media Cybernetics, Bethesda, MD). The data are expressed as the percentage of the level of density induced by TNF-α (set at 100%). All experiments were performed at least in triplicate (n = 3).

### Flow cytometric analysis

Expression of MAdCAM-1 analysis was performed by flow cytometry with FACScan (Becton Dickinson, Mountain View, CA) with the program CELL Quest (Becton Dickinson). For MAdCAM-1 protein expression, HEC(5 × 10^6^) were trypsinized, washed with PBS containing 0.5% BSA plus 0.1% NaN_3_, and 5% heat activated normal rabbit serum, re-suspended in the Washing Buffer(PBS containing 0.1% bovine serum albumin (BSA) and 0.1%NaN_3_), and incubated with fluorescein isothiocyanate-conjugated anti-MAdCAM-1 monoclonal antibody for 60 min at room temperature. The cells were washed three times with the Washing Buffer, re-suspended in PBS containing 1% BSA and 0.1%NaN_3_, and then fixed in PBS containing 4% formalin.

### TK-1 lymphocyte adhesion assay

Mouse CD8^+ ^T cell lymphoma tyrosine kinase (TK)-1 cells that constitutively express the integrin α_4_β_7 _[27] were kindly donated by Dr. Eugene Butcher (Stanford Univ., CA). These cells were cultured in RPMI medium supplemented with 10% FCS, 2 mM L-glutamine, and 0.05 mM 2-mercaptoethanol. Briefly, TK-1 cells were suspended in culture medium and fluorescence labeled by incubating TK-1 cells at 2 × 10^6 ^cells/ml with 0.02 mg calcein AM (Sigma) at 37°C for 15 min. The cells were then washed twice with ice-cold HBSS, spun at 250 g for 5 min to remove unincorporated fluorescent dye, and finally re-suspended in HBSS. The TK-1 lymphocyte cell line used in this assay expresses high levels of the α4β7 integrin [9,28], which can interact with multiple ligands including mucosal addressin-1 (MAdCAM-1), and also VCAM-1, L-selectin and fibronectin [29]. HEC were grown in 96-well plates as described, and monolayers activated by incubation with TNF-α (20 ng/ml) for 24 h. Cytokine treated HEC endothelial cells were then washed three times with media, and labeled TK-1 cells then added to 5:1 lymphocyte to endothelial cell ratio [30]. Cells were allowed to bind for 15 min under static conditions. At the end of the incubation period, the monolayers were washed twice with HBSS and plates read on a Fluoroskan Ascent (Labsystems, Helsinki, Finland) using excitation at 485 nm, and emission at 515 nm. Blank wells that did not contain labeled TK-1 cells were run as 0% TK-1 adhesion controls. The data are expressed as a percentage of TNF-α-induced (maximal) level of fluorescence. In each protocol, treatments were performed in triplicate.

### Analysis of nitric oxide donors and cell redox in TNF-α-induced MAdCAM-1 expression

DETA-NO and SperNO were purchased from Alexis corp. (San Diego, CA). L-NAME, NAC (N-acetylcysteine) were purchased from Sigma (St. Louis, MO). To examine NO effect for induction of MAdCAM expression, HEC were pretreated with drugs 1 h before TNF-α stimulation. HEC were harvested 24 h after TNF-α treatment and were Western blotted as described.

### Statistical analyses

All values are expressed as mean ± SD. Data were analyzed using one-way analysis of variance with Bonferroni post-testing for multiple comparisons. Probability (*P*) values of < 0.05 were accepted as significant.

## Results

### Hepatic endothelial cells

**Figure 1:**
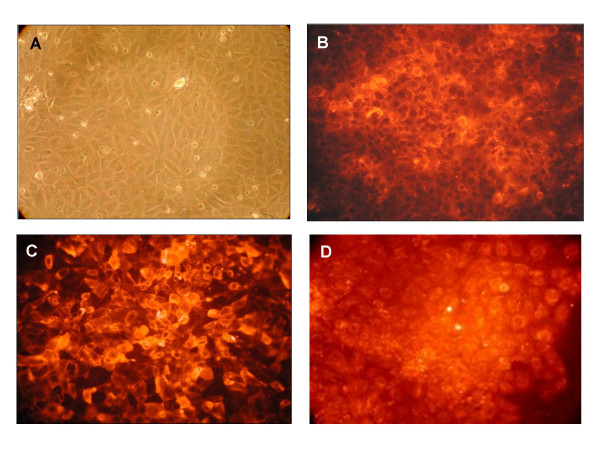
Establishment of hepatic endothelial cells (HEC). A. Phase contrast image of HEC. B. Immunofluorescent staining for VCAM-1 in HEC. C. Immunofluoresent images of mouse endothelial cell antigen-32 (MECA-32) expressed on the surface of HEC monolayers. D. Distribution of DiI-labeled acetylated low density lipoprotein -(LDL), an endothelial cell specific biomarker incorporated into endothelial cells by receptor mediated endocytosis.

**Figure 2:**
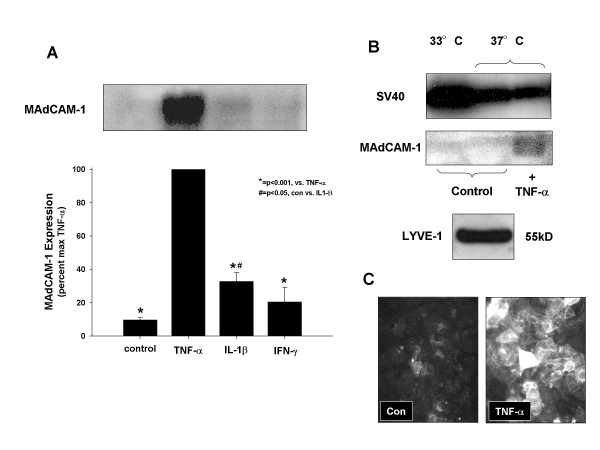
MAdCAM-1 is expressed by cytokine activated hepatic endothelial cells. A. Hepatic endothelial cells were treated with TNF-α (20 ng/ml), or IL-1β (10 ng/ml) or IFN-γ (1000 u/ml) for 24 h. B. Immunoblotting for SV40 Large-T antigen and MAdCAM-1 C. Immunostaining for MAdCAM-1 on the HEC cell surface.

The hepatic endothelial cells (HEC) exhibited a typical cobblestone morphology (see Figure 1A). All cells immunostained positively for VCAM-1 and anti-MECA32 (see Figures 1B, 1C). HEC cells took up DiI-labeled acetylated low density lipoprotein (AcLDL) (see Figure 1D), markers expressed by endothelial cell cultures *in vitro*. These cells also express lymphatic vascular endothelial vascular endothelial hyaluronan receptor-1 or LYVE-1, (see Figure 2B), which has been used to identify sinusoidal endothelial cells [31]. Therefore, our results are consistent with these HEC as pure endothelial cells with characteristics of *sinusoidal *hepatic endothelial cells.

### Analysis of MAdCAM-1 expression on hepatic endothelial cells

We examined MAdCAM-1 expression 24 h after stimulation with cytokines (TNF-α, IL-lβ, IFN-γ) Expression of MAdCAM-1 protein was measured by immunoblotting (using MECA-367 mAb). MAdCAM-1 was not constitutively expressed on unstimulated HEC but was induced by TNF-α (20 ng/ml) (see Figure 2A). IL-1β (10 ng/ml) also induced MAdCAM-1, but to a lesser extent than TNF-α. MAdCAM-1 was not expressed in response to IFN-γ. Next, we examined SV40 activity in response to the '*permissive*' temperature and TNF-α. We showed SV40 activity to a lesser extent after changing cells to 37°C, but there was no effect with regard to the TNF-α response (see Figure 2B). Also, we showed that MAdCAM-1 was present on the external endothelial cell surface based on immunostaining of the cell surface without prior permeabilization (see Figure 2C).

### TNF-α stimulates MAdCAM-1 expression dose-and time- dependently

**Figure 3:**
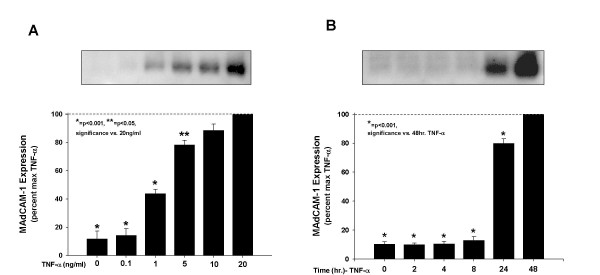
TNF-α stimulates MAdCAM-1 expression in a dose-and time- dependent manner. A. Confluent HEC endothelial cells were incubated for 24 h with increasing concentrations of mouse TNF-α (as indicated), and MAdCAM-1 induction (measured as western blotting density), expressed as a percent of the maximum, (set as the scan density in 24 h, 20 ng/ml TNF-α treated HEC, [100%]). MAdCAM-1 Expression is TNF-α concentration dependent B. HEC were incubated with 20 ng/mL TNF-α for time periods up to 48 h (as indicated). MAdCAM-1 protein expression was time dependently increased at 12, 24 and 48 h. (Max expression [100%] in these experiments was determined as the blotting scan density seen at 48 hours). Expression at T = 0 represents MAdCAM-1 expressed by un-stimulated hepatic endothelial cells. Each value represents the mean ± s.e.; each group (n = 3). *P < 0.01, **P < 0.01 vs. TNF-α treatment.

In controls, MAdCAM-1 was only faintly detected. Incubation of HEC with TNF-α for 24 h caused a dose-dependent increase in MAdCAM-1 (see Figure 3A). MAdCAM-1 was significantly increased by TNF-α (>2.0 ng/ml); maximum MAdCAM-1 expression occurred at 20 ng/ml. TNF-α (20 ng/ml) for 8, 24 or 48 h caused a time-dependent increase in MAdCAM-1; maximum MAdCAM-1 expression was observed at 48 h-α (see Figure 3B).

### MAdCAM-1 expression in HEC after TNF-α stimulation

**Figure 4:**
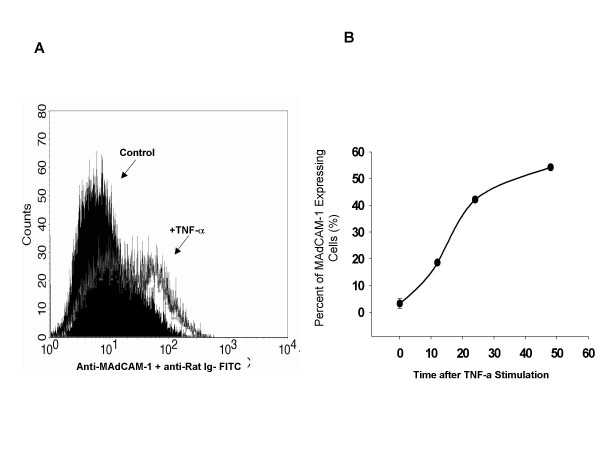
Quantitation of cells expressing MAdCAM-1 protein measured by flow cytometry A. MAdCAM-1 expression 24 h after TNF-α stimulation (20 ng/ml) B. Time course analysis of MAdCAM-1 expression. The concentration of TNF-α in this study was 20 ng/ml. Values represent means ± SE; n = 3 experiments in each group.

To quantify the amplitude of MAdCAM-1 signal and the percentage of cells that are positive after treatment with different cytokines and time course, we analyzed MAdCAM-1 expression after TNF-α stimulation by FACS. Figure 4A shows that 43% of the cells expressed MAdCAM-1 for 24 h. MAdCAM-1 expression is time-dependent, but the maximum expression is 53% after 48 h (see Figure 4B).

### NO reduces MAdCAM-1 expression on hepatic endothelial cells

**Figure 5:**
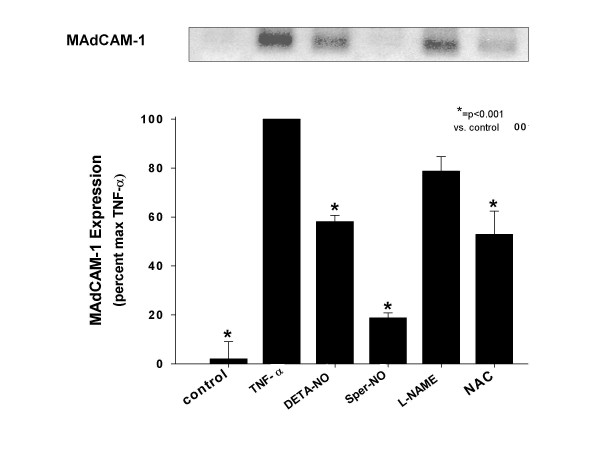
Effect of nitric oxide (NO) donors and N-acetylcysteine, an antioxidant on MAdCAM-1 expression by HEC in response to TNF-α. HEC were pretreated for 30 minutes with the NO donors, DETA-NO or SperNO (500 uM), or L-NAME (1 mM), or N-acetylcysteine (1 mM). Tumor necrosis factor (TNF)-α (20 ng/ml) was then added to these cultures for 48 h. MAdCAM-1 expression was significantly increased by TNF-α and significantly reduced by DETA-NO, SperNO or NAC, but not by L-NAME. Values represent the mean ± SE; n = 3 experiments in each group. * = p < 0.01 vs. TNF-α treatment.

To determine the effect of NO on TNF-α-induced MAdCAM-1 expression, 500 μM DETA Neonate, 500 μM Spermine NONOate, 1 mM N-acetyl cysteine (NAC), and 1 mM L-NAME were incubated with HEC 1 h before TNF-α stimulation. NO donors (DETA NONOate, spermine NONOate) significantly decreased TNF-α-induced MAdCAM-1 expression (see Figure 5). The non-selective NO synthase inhibitor (L-NAME) failed to limit MAdCAM-1 expression. On the other hand, the antioxidant NAC reduced MAdCAM-1 expression (see Figure 5).

### MAdCAM-1 expressed in HEC is functional

**Figure 6:**
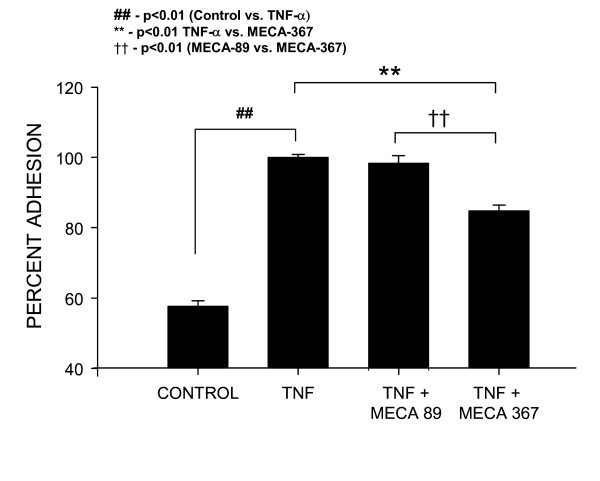
TNF-α induces MAdCAM-1 dependent adhesion of TK-1 lymphocytes to HEC. Compared to controls which exhibited 57.68 ± 3.77% of the maximum lymphocyte adhesion, 20 ng/ml TNF-α (24 h), induced a significant increase in TK-1 cell adhesion (set as maximal '100%' adhesion). The increased adhesion induced by TNF-α was significantly reduced to 84.8 ± 3.99% of maximal adhesion by pre-/co-treatment of HEC with the blocking mouse MAdCAM-1 Ab 'MECA-367'(10 ug/ml). The binding but not blocking MAdCAM-1 antibody MECA-89 did not significantly reduce TNF-α induced adhesion (98 ± 5.25% maximal adhesion). Each value represents the mean ± s.d.; each group (n = 6).

Having established several signals in the regulation of MAdCAM-1 expression by hepatic endothelial cells, we next examined lymphocyte adhesion after cytokines using the mouse lymphocyte cell line TK-1. Figure 6 shows that control adhesion of TK-1 cells was about 57.68 ± 3.77 of the maximal adhesion induced by TNF-α. TNF-α induced maximal adhesion (100 ± 2.1%) which was significantly elevated compared to controls (p < 0.01TNF-α vs. control). This level of adhesion was inhibited by pre-incubation of hepatic endothelial cells with the blocking anti-MAdCAM-1 antibody MECA-367 (10 ug/ml). This antibody reduced TK-1 adhesion to 84.8 ± 3.99% of TNF-α (*p *< 0.01 TNF-α + MECA-367 vs. TNF-α). Similar studies performed using MECA-89, a MAdCAM-1 binding, but not blocking MAdCAM-1 antibody [32], did not significantly reduce TK-1 cell binding following TNF-α (98 ± 5.24% of the maximum adhesion induced by TNF-α).

## Discussion

The present study demonstrates that MAdCAM-1 is induced on HEC on exposure to some cytokines (TNF-α and IL-1β), and that this MAdCAM-1 can support lymphocyte adhesion (since anti-MAdCAM-1 mAb blocked TNF-α stimulated adhesion). This indicates that MAdCAM-1 might help regulate lymphocyte adhesion/extravasation in the liver during inflammation. MAdCAM-1 is an endothelial CAM of the immunoglobulin superfamily (along with ICAM-1 and VCAM-1) that has been implicated in the selective recruitment of lymphocytes to sites of inflammation in the gut [2,3]. Clinical studies showed that E-selectin and VCAM-1, (which were absent in normal human liver tissue), became strongly expressed in inflammatory liver disease [33]. With respect to MAdCAM-1, it is known that MAdCAM-1 was expressed on portal vein and sinusoidal endothelium in chronically inflamed human liver [34]. Grant et al. showed that MAdCAM-1 in the liver was functionally active because α_4_β_7_+ T cells were found to bind with MAdCAM-1-expressing vessels and circulating T cells from patients with inflammatory bowel disease bound to human hepatic endothelium via a MAdCAM-1 dependent pathway [7]. Our data support that idea that MAdCAM-1 is expressed on the sinusoidal endothelial cells of the liver.

Pathologically, acute and chronic human hepatitis is also characterized by prominent infiltration of lymphocytes (mainly T cells) into the parenchyma and perivascular interstitial tissues of the liver [35]. Lymphocyte attachment to endothelium within central and portal veins may provoke what is termed "endothelialitis" [36], and lymphocyte migration into the sub-endothelial layer is common in inflammatory liver disease [37]. T-cell mediated hepatitis can be induced in mice by administration of Con A [38]. In this model, after endothelial adhesion, lymphocytes promptly migrate out of vessels and accumulate in the perivascular areas (such as the space of Disse), perivenular intestinal tissue, and portal tract. TNF-α and IFN-γ released from activated T cells play important roles in the development of this order. T-cell cytotoxicity mediated by Fas-Fas ligand [39] or perforin [40] might also be involved in hepatic injury. Although the main infiltrating cells in this murine hepatitis model are non-specifically activated lymphocytes directed to infected hepatocytes (as seen in human viral hepatitis), the main pathological features (i.e., massive hepatocellular degeneration and prominent T-cell infiltration) [38] are shared by these two. This indicates that lymphocyte adhesion/transmigration is commonly seen in various kinds of hepatic inflammation. We found that the TK-1 lymphocyte line bound to HEC via α_4_β_7 _binding to MAdCAM-1.

Primary sclerosing cholangitis (PSC) and primary biliary cirrhosis (PBC) are both chronic cholestatic liver diseases with progressive destructive inflammatory fibrosis of intrahepatic bile ducts, especially the septal and interlobular bile ducts. While the etiology of both diseases is unknown, there is evidence to suggest that immunological mechanisms may be important [12]. Abnormalities in lymphocyte subsets exist in peripheral blood and liver in both PSC [41] and PBC [42]. Infiltrating T cells in both of these models are closely associated with areas of bile duct destruction [41,43]. Grant et al. reported that vessels in the human liver support adhesion of α_4_β_7_+ mucosal lymphocytes via binding to expressed MAdCAM-1 on liver endothelium and proposed a mechanism to explain the hepatic recruitment of mucosal lymphocytes in inflammatory liver disease complicating IBD [7]. Eksteen et al. proposed that long-lived populations of memory lymphocytes arise as a consequence of bowel inflammation and that these cells express homing receptors that direct their subsequent migration not only to the gut but also to the liver [44]. We already reported that MAdCAM-1 on colonic endothelial cells after TNF-α stimulation *in vitro *[45]. Our results here may support previous assumptions that MAdCAM-1 expression might be one of the causes of lymphocyte-mediated liver disease, like autoimmune hepatitis and primary sclerosing cholangitis, which complicate inflammatory bowel disease.

NO is an important modulator of adhesion molecule expression in both acute and chronic inflammatory states and may influence the course of chronic inflammation. It is known that NO can function both as an oxidant or antioxidant depending on the availability of reactive oxygen species. Similarly, the concentration of NO can also augment or inhibit oxygen-radical mediated tissue damage and lipid peroxidation. It has also been reported that inhibition of endothelial nitric oxide synthases (using the non-selective NOS inhibitor L-NAME) induces endothelial adhesion molecules (ICAM-1 and VCAM-1) in HUVEC [17]. Ischemia/Reperfusion (I/R) is a consequence of liver transplantation and resectional surgery, as well as hemorrhagic shock and thermal injury. The role that NO plays in this process has been the subject of active debate. A growing body of experimental evidence suggests that NO may also modulate I/R-induced tissue injury in various organ systems [17,19]. *In vitro *and *in vivo *data show that NO protects tissue by decomposing superoxide radical (O_2 _^-^) [46]. It has also been suggested that NO modulates the activity of transcription factors such as NF-kB [47]. Although some studies demonstrate that NO limits or down-regulates I/R-induced liver injury, other reports suggest that NO promotes I/R-induced hepatocellular damage, possibly due to the formation of very strong oxidizing species like peroxynitrite (ONOO^-^) [48]. Much of this controversy is probably due to the wide use of non-specific inhibitors of the different NOS isoforms [48]. With the advent of genetically engineered mice, it is now possible to more precisely examine the role of NO in I/R-induced liver injury [46,49]. Hines et al. have shown that endothelial nitric oxide synthase (e-NOS)- and inducible nitric oxide synthase (iNOS)-deficient mice are highly susceptible to damaging effects of liver I/R [50]. They have also shown that eNOS-, but not iNOS, derived NO modulates the expression of pro-inflammatory cytokines possibly limiting the observed tissue injury with a marked up-regulation in eNOS message during I/R potentially a protective mechanism within the liver. In this present study, we investigated how MAdCAM-1 expression in HEC was altered by NO donors and synthase inhibitors. We observed that both a short and long-acting NO donor significantly reduced TNF-α-induced expression of MAdCAM-1 expression in hepatic endothelial cells. The effects of NO donors appear to reflect their ability to prevent the nuclear translocation of NF-kB. The MAdCAM-1 promoter has several NF-kB binding sites [51], and is necessary to induce MAdCAM-1 expression. Both a short and long acting NO donor blocked MAdCAM-1 induction, but the slow-releasing NO DETA-NO was about 5 times more effective than SperNO (on a molar basis). This probably reflects a requirement for the continuous production of NO which is needed to block cytokine signaling in this system. Oshima et al. reported that in the SVEC-based experiments (a cell line used to study mechanisms of MAdCAM induction) that NO limits inflammation via NF-kB inhibition, since NO donors effectively block p65 translocation into the nucleus [52].

Cellular thiol status has been shown to modulate transcription factor activation of gene expression mediated TNF-α, IL-1, LPS, or H_2_O_2_. Staal et al. demonstrated that low cell thiol levels promote NF-kB activation, whereas exogenous cysteine and N-acetyl-L-cysteine (NAC) inhibit NF-kB activity [53]. Moreover, a decrease in GSH induced by inhibition of GSH biosynthesis was shown to alter the NF-kB activation responses to LPS [54] or TNF-α [55]. Samarasinghe et al. showed that sinusoidal endothelial cells undergo significant intracellular oxidative stress following re-oxygenation, and their viability is critical related to cell GSH levels [56]. Similarly, we show here that pretreatment with NAC inhibited cytokine induced MAdCAM-1 expression.

MAdCAM-1 was induced on HEC by TNF-α and IL-1β (but not by IFN-γ). Similarly, HEC adhered lymphocytes after TNF-α stimulation, and this adhesion was inhibited by anti-MAdCAM-1 antibody treatment. This suggests that lymphocytes are recruited and bound to HEC via MAdCAM-1. This type of adhesion might be a contributing mechanism in several inflammatory liver diseases, including viral-induced liver disease, autoimmune liver disease, and I/R induced liver injury. We believe that this HEC cell system may represent a useful tool for modeling several acute and chronic liver diseases *in vitro*.

## List of abbreviations

HEC, hepatic endothelial cells; MAdCAM-1, Mucosal addressin cell adhesion molecule-1; ICAM-1, intercellular adhesion molecule-1; ICAM-1, intercellular adhesion molecule-2; VCAM-1, vascular cell adhesion molecule-1 (VCAM-1) PSC, ECAMs, endothelial cell adhesion molecules; primary sclerosing cholangitis; PBC, primary biliary cirrhosis; NAC, N-acetyl cysteine; I/R, ischemia and reperfusion; TK, tyrosine kinase; AcLDL, acetylated low density lipoprotein (AcLDL); ROS, reactive oxygen species; e-NOS, endothelial nitric oxide synthase (e-NOS); iNOS, inducible nitric oxide synthase; ONOO^-^, peroxynitrite; IBD, inflammatory bowel disease

## Competing interests

The author(s) declares that there are no competing interests.

## Authors' contributions

TA designed and executed the majority of these studies.

RRL prepared the cells used in this study and helped design adhesion protocols.

YW participated in the study design and studies involving LYVE-1.

PAJ participated in the study design and revisions.

AM participated in study design and statistics.

JSA conceived of the study and participated in its design and coordination, data interpretation and revisions.

MHJ performed additional western blotting and adhesion studies
